# N-Terminal Domain Mediated Regulation of RORα1 Inhibits Invasive Growth in Prostate Cancer

**DOI:** 10.3390/ijms20071684

**Published:** 2019-04-04

**Authors:** Su Chan Park, Il-Geun Park, Hyunkyung Kim, Ji Min Lee

**Affiliations:** 1 Department of Molecular Bioscience, College of Biomedical Sciences, Kangwon National University, Chuncheon 24341, Korea; psc4932@naver.com (S.C.P.); dlfrms2382@naver.com (I.-G.P.); 2 Center for Theragnosis, Biomedical Research Institute, Korea Institute of Science and Technology, Seoul 02792, Korea; hkk@kist.re.kr

**Keywords:** RORα1, Wnt/β-catenin pathway, prostate cancer, NTD

## Abstract

Four members of the retinoic acid-related orphan receptor α (RORα) family (RORα1, RORα2, RORα3 and RORα4) are transcription factors that regulate several processes including circadian rhythm, lipid metabolism, cerebellar development, immune function, and cancer. Only two isoforms, RORα1 and 4, are specifically co-expressed in the murine and human. In the present study, we identified a specific N-terminal domain (NTD) of RORα1 that potentiated the downregulation of target genes involved in tumor progression and proliferation, based on results from RORα-deficient mouse embryonic fibroblasts and prostate carcinoma tissues. The hyperactivation of proliferative target genes were observed in RORα-deficient embryonic fibroblasts, and reconstitution of RORα1 inhibited this activation by a NTD dependent manner. Downregulation of RORα1 and upregulation of Wnt/β-catenin target genes were correlated in prostate cancer patients. These findings revealed the control of invasive growth by NTD-mediated RORα1 signaling, suggesting advanced approaches for the development of therapeutic drugs.

## 1. Introduction

Defining the molecular strategies by which distinct signaling pathways control downstream target genes coordinately is central for understanding development, disease, and homeostasis. The Orphan nuclear receptor (ONR) family perform vital roles in controlling subsets of target gene expression both positively and negatively in diverse signaling pathways in the regulation of disease, neurogenesis, and homeostasis [1,2]. ONRs are ligand-activated transcription factors, for which no cognate ligands have been identified thus far. Similar to the other members of nuclear receptors, ONRs are composed of two conserved structural domains, namely, a DNA-binding domain (DBD) and a ligand-binding domain (LBD) [3,4]. The DBD is composed of two zinc finger motifs that provide links for DNA-protein and protein-protein interactions. The LBD is also conserved among nuclear receptors and functions as a domain for ligand binding, receptor dimerization, and transcriptional activation/repression. It has been reported that the function of the N-terminal domain (NTD), which is not well conserved among the receptors, is to confer different DNA-binding properties to various transcription factors in a cell- and context-dependent manner [5]. The retinoic acid receptor (RAR)-related orphan receptor α (RORα) has four isoforms referring to RORα1 to 4 in human, with distinct NTD but identical DBD and LBD regions, by alternative splicing mechanism [5,6,7]. RORα has been reported to bind promoter as monomers or homodimers to binding elements composed of a 6-bp AT-rich sequence prior to a core motif PuGGTCA (RORE), and RORα-binding sites have been demonstrated for numerous genes, including *Shh, Slc1a6, N-myc*, *Cav3, Cpt1, Pcp2, Pcp4* and *Crygf* [8,9,10,11,12]. Furthermore, RORα has been implicated in many pathophysiological processes including circadian rhythm, immunity, metabolic pathways and cancer [13,14,15,16,17].

The crosstalk between the nuclear receptor family and the Wnt/β-catenin pathway is emerging as a clinically and biologically significant issue [18,19]. Given that nuclear receptors and their cognate ligands play roles as active regulators of normal physiology as well as tumor pathologies, the nuclear receptors can functionally collaborate with dominant tumorigenic pathways, for example, the Wnt signaling [20,21,22]. This binding might drive changes in cellular adhesion and tumorigenesis. Wnt ligands and β-catenin signaling are potent initiators of oncogenesis in that the mutation of these molecules provides a predictor of cancer progression [23,24,25,26]. The approaches that demonstrate the cross-regulation of Wnt signaling with that of ONRs could provide a platform on which potential changes in cell adhesion and the transcription profile during cancer progression beyond colorectal cancers can be evaluated.

Canonical Wnt signaling causes β-catenin accumulation with T cell factor (TCF)/lymphoid enhancer factor (LEF) in the nucleus; which then regulates target gene expression [27,28]. In the absence of Wnt binding to frizzled, the level of β-catenin in the cytoplasm remains low due to the degradation of β-catenin by 26S proteasome after paired phosphorylation through the serine/threonine kinases casein kinase I (CKI) and glycogen synthase kinase-3β (GSK-3β) [29,30]. The activation of dishevelled (Dvl) inhibits GSK-3β, resulting in the accumulation of cytoplasmic β-catenin, which triggers the translocation of β-catenin to the nucleus for the increasing target gene expression. Emerging evidence suggests that β-catenin is associated with colorectal cancers [31]. However, it is now evident that β-catenin is also essential in breast and prostate cancers [32,33]. Therefore, we investigated a specific cross-regulation between RORα1, not by RORα4, and Wnt/β-catenin signaling in a prostate cancer cell culture model, a *ROR*α-deficient mouse model, and in clinical samples of patients with prostate carcinomas.

## 2. Results

### 2.1. Selective Linkage of RORα1, not RORα4, in the Downstream Signaling Pathway

**Figure 1:**
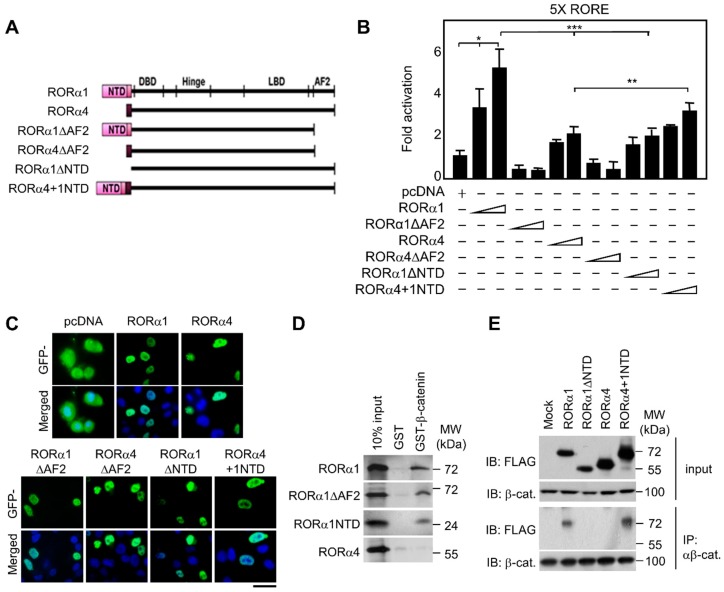
N-terminal domain of RORα1 is sufficient for regulating target genes. (**A**) Illustration of the structure of RORα1, RORα4, RORα1ΔAF2, RORα4ΔAF2, RORα1ΔNTD, and RORα4 + 1NTD. (**B**) Luciferase assay was conducted using FLAG-pcDNA, RORα1, RORα4, RORα1ΔAF2, RORα4ΔAF2, RORα1ΔNTD, and RORα4 + 1NTD on a 5× RORE luciferase reporter. Values are expressed as mean ± SD for three independent experiments and normalized by β-galactosidase expression (mean ± SD, *n* = 3). The p value was calculated by a *t*-test (* *p* < 0.05, ** *p* < 0.01) or one-way ANOVA (*** *p* < 0.001). (**C**) Subcellular localization of GFP-pcDNA, GFP-RORα1, GFP-RORα4, GFP-RORα1ΔAF2, GFP-RORα4ΔAF2, GFP-RORα1ΔNTD, and GFP-RORα4 + 1NTD constructs (green). Nuclei were visualized by DAPI staining (blue). Scale bar, 20 μm. (**D**) Interaction of each in vitro transcribed and translated RORα1, RORα1ΔAF2, RORα1NTD and RORα4 constructs with glutathione S-transferase (GST) or GST fusion of β-catenin was assessed by GST pulldown assay. (**E**) Coimmunoprecipitation of endogenous β-catenin with each FLAG-tagged RORα construct.

We previously reported that β-catenin provided a link with RORα for coordinating the expression of genes required for cerebellar development [9,19]. Since RORα4 does not possess the NTD of RORα1, we utilized chimeric RORα1/RORα4 proteins (RORα4 + 1NTD) in which the NTD of RORα1 was fused to RORα4 (Figure 1A). Compared to RORα1, RORα1ΔNTD or RORα4 exhibited significantly diminished transcriptional activation on the RORE-luciferase reporter (*p* value < 0.001), whereas RORα4 + 1NTD permitted the transcriptional activation function comparable to that of RORα1 (Figure 1B). Immunohistochemical studies revealed that all of the tested constructs showed an almost exclusive nuclear staining pattern, illustrating that the difference in transcriptional activation mediated by each RORα construct was not due to different subcellular localization (Figure 1C and Figure S1). Both GST-pulldown and immunoprecipitation assays confirmed the selective binding of β-catenin to RORα1 (Figure 1D). RORα4+1NTD construct restored the binding ability to β-catenin, suggesting the NTD of RORα1 indeed functions as a molecular determinant to confer the functional link to β-catenin signaling (Figure 1E). In support of this idea, β-catenin potentiated RORα1-dependent transcriptional activation, whereas β-catenin failed with RORα1ΔNTD or RORα4 (Figure S2). For the nuclear receptor and ONR, it is widely accepted that the ligand-dependent transcription activation capacity is mediated via the activation function AF2 in the LBD. However, for the RORα1 a different mechanism of selective activation seems to exist, where the NTD harbors a strong transactivating function with β-catenin. Together, the coactivator function of β-catenin on RORα target gene promoters requires the binding of β-catenin to RORα1 via the NTD of RORα1.

### 2.2. Wnt Signaling Is Hyperactivated in RORα-Null Mouse Embryonic Fibroblasts

**Figure 2:**
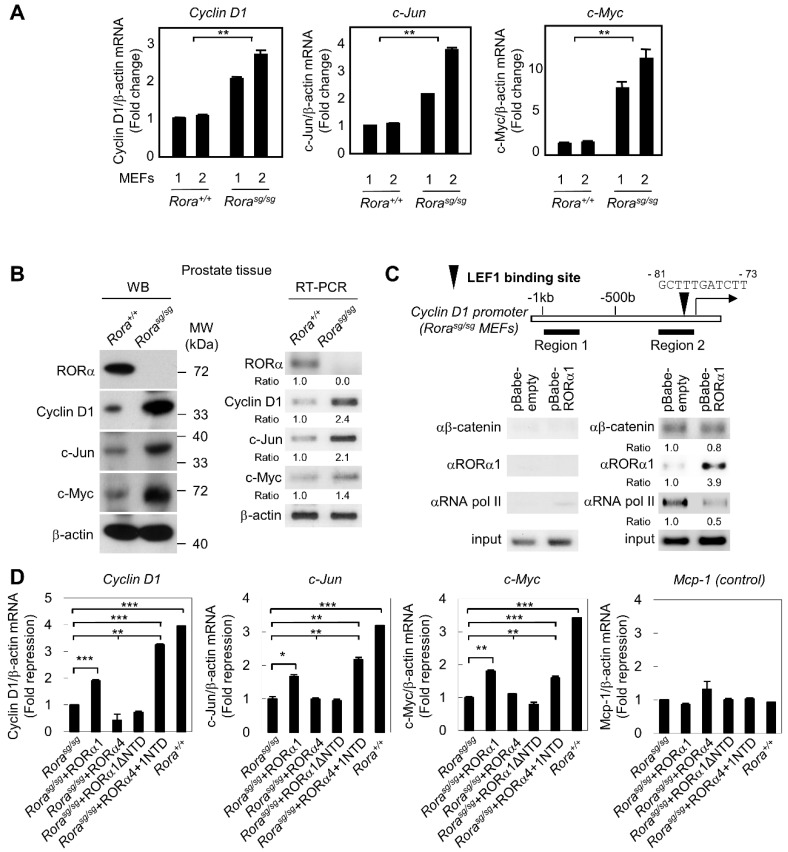
RORα1 transcriptional repressive function on Wnt target genes in MEFs. (**A**) Real-time quantitative RT-PCR analysis of *cyclin D1*, *c-jun*, and *c-myc* was performed on primary mouse embryonic fibroblasts prepared from two independent *Rora^+/+^* (*Rora^+/+^* 1 and *Rora^+/+^* 2) or *Rora^sg/sg^* (*Rora^sg/sg^* 1 and *Rora^sg/sg^* 2) mice (mean ± SD, *n* = 3). The p value was calculated by one-way ANOVA (** *p* < 0.01). (**B**) Immunoblotting and RT-PCR analyses of RORα1 and Wnt target genes in prostate tissue from *Rora^+/+^* and *Rora^sg/sg^* mice. (**C**) ChIP assay on the *cyclin D1* promoter from *Rora^sg/sg^* MEFs. Cells were either infected with control retrovirus (pBabe-empty vector) or infected with retrovirus expressing RORα1 (pBabe-RORα1). Occupancy of the *cyclin D1* promoter by β-catenin, RORα1, and RNA polymerase II is indicated. The upper illustration represents the location of the LEF1 binding site on the *Cyclin D1* promoter. The proposed model of RORα1 serving as a co-repressor for β-catenin transcriptional activity on the promoter of the target gene, *Cyclin D1* (right panel). (**D**) Real-time quantitative RT-PCR analysis of *cyclin D1*, *c-jun*, *c-myc, and Mcp-1* was performed on *Rora^sg/sg^*, *Rora^sg/sg^* reconstituted with RORα1, RORα4, RORα1ΔNTD, RORα4 + 1NTD, and *Rora^+/+^* MEFs (mean ± SEM, *n* = 3). The p value was calculated by a t-test (** *p* < 0.01, *** *p* < 0.001) or one-way ANOVA (** *p* < 0.01).

The data obtained from a NTD-mediated target gene regulation allowed us to further investigate whether the loss of RORα was responsible for the hyperactivation of Wnt target genes by using primary mouse embryonic fibroblasts (MEFs) prepared from *ROR*α-deficient *staggerer* (*Rora^sg/sg^*) mice, which had a spontaneous loss-of-function mutation in the *ROR*α gene [10]. LiCl induced Wnt target genes such as *cyclin D1*, *c-jun*, and *c-myc*, and quantitative RT-PCR analysis revealed that Wnt target genes in *ROR*α-deficient fibroblasts were hyperactivated when compared with those in wild-type (*Rora^+/+^*) fibroblasts (Figure 2A). Increased expression levels of both proteins and RNAs of cyclin D1, c-jun and c-myc in the prostate tissues from *Rora^sg/sg^* mice were observed compared to those in *Rora^+/+^* mice by immunoblotting and RT-PCR analyses (Figure 2B). These results strongly supported our hypothesis that RORα1 plays a crucial role in the downregulation of Wnt target genes in vivo.

Furthermore, we performed a retroviral reconstitution experiment with each retroviral RORα construct into *Rora^sg/sg^* MEFs. ChIP assay unveiled that recruitment of RORα1 to the *cyclin D1* promoter with decreased Pol II levels on the target gene (Figure 2C). Real-time RT-PCR analysis revealed that reconstitution of RORα1 suppressed the expression of *cyclin D1*, *c-jun*, and *c-myc* in *Rora^sg/sg^* MEFs, whereas introduction of either RORα4 or RORα1ΔNTD did not alter the transcript levels of Wnt target genes significantly (Figure 2D). These data confirmed that Wnt target genes were indeed hyperactivated in *ROR*α-null mouse embryonic fibroblasts and prostate tissues. Based on the studies involving the *ROR*α-deficient embryonic fibroblasts and prostate tissues from *Rora^sg/sg^* mice, we concluded that RORα1 induced the repression of Wnt target genes such as *cyclin D1*, *c-jun*, and *c-myc*.

### 2.3. RORα1 Specifically Inhibits Cell Proliferation via the Downregulation of Wnt Target Genes

**Figure 3:**
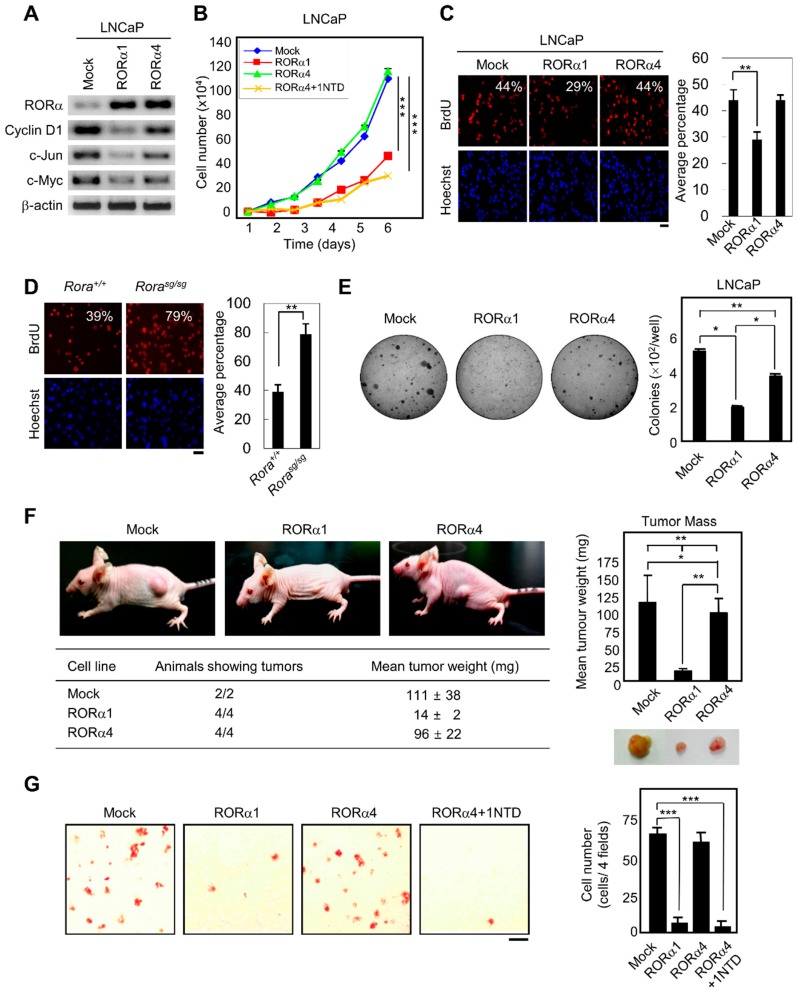
RORα1-mediated inhibition of in vivo tumorigenesis and metastatic potential. (**A**) RT-PCR analysis of the Wnt target gene transcripts after overexpressing either RORα1 or RORα4 in LNCaP cells. (**B**) Proliferation curves of RORα1, RORα4 or RORα4 + 1NTD-expressing LNCaP cells. Values are represented as mean ± SD for three independent experiments (mean ± SD, *n* = 3). The p value was calculated by a *t*-test (*** *p* < 0.001). (**C** and **D**) BrdU incorporation in LNCaP, RORα1, RORα4-expressing LNCaP cells (**C**) and *Rora^+/+^*, *Rora^sg/sg^* MEFs (**D**) (Red fluorescence, left) compared to the total number of nuclei (Hoechst staining, right). The average percentage of BrdU incorporated cells from three different fields is indicated (mean ± SD, *n* = 3). The p value was calculated by a *t*-test (** *p* < 0.01). Scale bar, 20 μm. (**E**) The anchorage-independent growth of LNCaP cells expressing RORα1 or RORα4 in soft agar. Values are expressed as mean ± SEM for two experiments in 6-well plates. The representative image is shown for each group. The p value was calculated by a t-test (* *p* < 0.05) or one-way ANOVA (** *p* < 0.01). (**F**) Subcutaneous tumor growth of LNCaP cells expressing RORα1 or RORα4 in nude mice. Mice were injected subcutaneously at the left flank with 1 x 10^7^ LNCaP (*n* = 2), RORα1 (*n* = 4) or RORα4 expressing cells (*n* = 4). Wet weight of tumors derived from LNCaP, RORα1 and RORα4 cells is shown. Tumor weight was calculated as described in Methods, four weeks after the injection of cells. Values are expressed as mean ± SEM and the p value was calculated by a t-test (* *p* < 0.05, ** *p* < 0.01) or one-way ANOVA (** *p* < 0.01). (**G**) The invasive activity of LNCaP cells expressing RORα1, RORα4, or RORα4 + 1NTD assayed in Matrigel chambers. Bar graph shows the mean number of cells per filter, and the number of cells was counted in four different fields (mean ± SD, *n* = 3). The p value was calculated by a *t*-test (*** *p* < 0.001). Scale bar, 100 μm.

Since RORα1 reduced the expression of cyclin D1 and c-myc, we examined whether RORα1 could inhibit proliferation through the concomitant reduction of cyclin D1 and c-myc expression in prostate cancer cells. LNCaP cells express basal levels of RORα1 and higher levels of cyclin D1, c-myc, and c-jun; however in RORα1-expressing LNCaP cells, a marked reduction of endogenous cyclin D1, c-myc, and c-jun expression were observed (Figure 3A and Figure S3). Introduction of RORα4, which is deficient in β-catenin binding, failed to suppress Wnt target genes in LNCaP cells (Figure 3A). These results demonstrated that the downregulation of Wnt target genes was a direct effect of RORα1 binding and inhibition of β-catenin-mediated activation in prostate cancer cells.

As upregulation of cyclin D1, c-myc, and c-jun is correlated with cell growth and proliferation, we next explored if the expression of RORα1 could repress cellular proliferation. A proliferation assay, which measured the increase in cell number over five days for RORα1, RORα4, and RORα4 + 1NTD expressing LNCaP cells along with control LNCaP cells revealed that NTD of RORα1 possessed a growth inhibitory function (Figure 3B). In support of the results shown in Figure 3B, knockdown of RORα1 by shRNA for RORα1 in RWPE1 normal prostate epithelial cells led to an increase in proliferation rate, confirming an inverse correlation between the proliferation rate and the RORα1 expression level (Figure S4). In light of such a compelling anti-proliferative effect of RORα1, we next determined whether RORα1 exerted its anti-proliferative effect by influencing DNA synthesis. Upon examination of BrdU incorporation, DNA synthesis in RORα1, but not in RORα4 cells was reduced compared to control LNCaP cells (Figure 3C). Furthermore, we observed a similar effect of RORα1 inhibiting DNA synthesis in MEFs. *Rora^sg/sg^* MEFs exhibited an increase in DNA synthesis compared to that of *Rora^+/+^* MEFs (Figure 3D).

We then examined other features known to be essential for cell growth and proliferation. As anchorage-independent growth is an essential property of cancer cell growth, we first tested whether RORα1, but not RORα4 could inhibit the colony-forming strength of LNCaP cells in soft agar. Consistent with the anti-proliferative properties of RORα1, LNCaP cells expressing RORα1 grew significantly slower than control cells (Figure 3E).

### 2.4. RORα1 Influences in Vivo Tumorigenesis and Metastatic Potential

Extending in vitro findings to an in vivo setting, the tumorigenesis experiments with LNCaP cell lines stably expressing RORα1 or RORα4 in athymic nude mice was examined. Tumors derived from RORα1, but not RORα4-expressing cells showed significantly reduced tumor weights compared to that obtained from control cells (Figure 3F). Collectively, these results suggested a mechanism intrinsic RORα1-mediated suppression of cellular proliferation, growth, and tumorigenesis in vivo was, at least in part, through the repression of Wnt target genes.

We further examined whether altering the RORα1 expression would not only influence tumor growth but also modulate metastatic potential of invasive prostate cancer cells using the Matrigel invasion assay (Figure 3G). Expression of RORα1 in LiCl-treated LNCaP cells significantly reduced invasion through Matrigel, whereas cells expressing RORα4 failed to reduce the invasive activity. Together, RORα1 expression regulated the invasive activity of tumor cells by inhibiting Wnt target genes involved in tumor progression, proliferation, and metastasis, and cross-regulation of RORα1 and the Wnt/β-catenin pathways were crucial for the modulation of invasive growth in prostate cancer cells.

### 2.5. Inverse Correlation of RORα1 and Wnt Target Genes in Prostate Carcinoma Tissues

**Figure 4:**
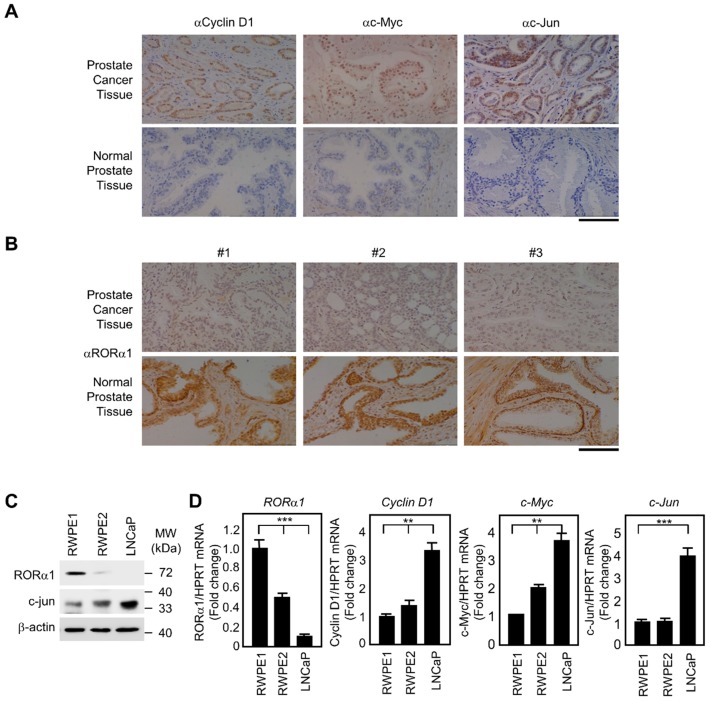
Tissue array and immunohistochemical analysis from prostate cancer patients. (**A**) Tissue samples from prostate cancer patients were stained with cyclin D1, c-myc, or c-jun antibodies along with normal prostate tissue samples. Scale bar, 100 μm. (**B**) Immunohistochemical staining of RORα1 was performed on the prostate carcinoma and normal tissues. Scale bar, 100 μm. (**C**) Expression of RORα1 and c-jun in prostate (RWPE1, RWPE2, and LNCaP) cell lines, as assessed by immunoblotting. (**D**) Real-time RT-PCR analysis of *ROR*α*1*, *cyclin D1*, *c-myc*, and *c-jun* transcripts in RWPE1, RWPE2, LNCaP cells (mean ± SD, *n* = 3). The *p* value was calculated by a t-test (*** *p* < 0.001) or one-way ANOVA (** *p* < 0.01, *** *p* < 0.001).

Constitutive activation of β-catenin signaling is involved in the development of human cancers, and overexpression of cyclin D1, c-myc, and c-jun, which are associated with tumor progression [34,35]. In contrast, little is known about the contribution of RORα1 to tumor suppression. Therefore, to ascertain whether our findings of cross-regulation of RORα1 and Wnt target genes in the cell culture and in *ROR*α-deficient fibroblasts do in fact corroborate, immunohistochemical staining was performed on the clinical samples of patients with prostate carcinomas. Immunohistochemistry experiments revealed a low expression of cyclin D1, c-myc, and c-jun in normal prostate tissues, and an upregulation of cyclin D1, c-myc, and c-jun in prostate carcinoma tissues (Figure 4A). The level of RORα1 inversely correlated with the expression of Wnt target genes in the human prostate tumor specimens (Figure 4B).

In support of this data in clinical samples, immunoblotting analysis revealed that RORα1 expression dramatically decreased in the prostate metastatic cancer cell line such as LNCaP compared to that in the normal prostate cell line such as RWPE1, while the expression of c-jun increased in LNCaP cells and decreased in RWPE1 cells (Figure 4C). The quantitative RT-PCR analysis also revealed that the RORα1 transcript was trans-repressed, and the *cyclin D1* and *c-jun* mRNA levels were upregulated in LNCaP cells (Figure 4D). Together, downregulation of RORα1 and upregulation of cyclin D1, c-myc, and c-jun were observed in prostate carcinoma tissues as well as in cancer cells.

## 3. Discussion

*Staggerer* mice, which have a spontaneous loss-of-function phenotype of the RORα gene, have been used for experimental studies for the past 40 years; however, these results have focused primarily on the investigation of cerebellar defects. *Staggerer* mice and *Rora^sg/sg^* mice exhibited similar phenotype and approximately 50% of the *Rora^sg/sg^* mice died shortly after weaning [36]. Our studies have broadened the spectrum of the action of RORα to the regulation of tumor progression and proliferation by an unanticipated cross-regulation with the Wnt/β-catenin pathway. RORα1 and RORα4 function as direct transcription factors on their binding promoters, however only RORα1 represses the activity of a second protein β-catenin through a protein-protein interaction on Wnt/β-catenin promoters. Since this trans-regulation acts between two different proteins, it is referred to as a trans-repressing process. Together with the results from *staggerer* mouse embryonic fibroblasts and prostate tissues, our study establishes a novel mechanism by which RORα1, but not RORα4, trans-represses the Wnt target genes by directly interacting with β-catenin via its NTD and thereby competing with other coactivators for binding to β-catenin.

In addition to defining combinatorial and coordinated control of the cross-regulation of RORα and Wnt/β-catenin signaling, our studies also suggest higher levels of modulation that serves to coordinate different signal transduction pathways in human disease. These approaches may provide a platform on which to evaluate changes in both the transcriptional profile and phenotype that can potentially occur during tumor progression and metastasis. RORα1 might be involved in the adjustment of biological physiologies by coordinating organized regulatory crosstalk between the Wnt/β-catenin pathway to integrate attenuated transcriptional activation to aggressive cancer progression and metastasis. Instead, it is envisaged that in a particular physiological environment, an upregulation or downregulation mechanism of RORα1 might be described, at least somewhat, by the administrative Wnt/β-catenin signaling pathways.

RORα has four different isoforms in human, whereas mice have only two isoforms, RORα1 and RORα4 by alternative splicing. The difference between NTD in RORα2 and RORα3 also confers different DNA binding specificities as well as transcriptional activities. Compared to RORα1 and RORα4, which show ubiquitous expression patterns in both humans and mice, RORα2 and RORα3 have tissue- and cell type-specific expression patterns only in humans. We previously reported the selective RORα2 roles in tumorigenesis via oncogenic target expression in human breast cancer [37]. In this study, we demonstrated the specific anti-tumor effect of RORα1 through novel mechanisms resulting in trans-repression of Wnt/β-catenin by NTD. Androgen receptor (AR) is one of the nuclear receptors and the major therapeutic target in aggressive prostate cancer. However, it has been reported that most initial responders to AR inhibitors alone in prostate cancer patients eventually develop acquired resistance. In order to circumvent the resistance to AR-targeted therapies, co-inhibition with the Wnt/β-catenin pathway inhibitor has been studied, as targeting β-catenin is critically involved in the development of resistance to AR-targeted therapies [38]. According to our results, we were able to demonstrate favorable attenuation of Wnt/β-catenin signaling by selective RORα1 activation, which could provide a better therapeutic option in prostate cancer.

The crosstalk between the Wnt/β-catenin pathway and the nuclear receptor family is emerging as a biologically significant area. Nuclear receptors are the primary drug targets used in various human diseases. The present results suggest an effective interrelating strategy for the therapeutic potentials of RORα1. Since we found that peptides corresponding to the NTD of RORα1 can block the Wnt/β-catenin signaling pathway, we suggest that this peptide may provide a potential reagent for identifying antagonists that might act to prevent diseases conceivably mediated by the Wnt/β-catenin pathway. Alternatively, it is conceivable that the development of therapeutic drugs that can trigger RORα1 level would be effective for targeting specific prostate cancers. The other possible binding partners of RORα1 via NTD underlying the anti-tumor effect of RORα1 warrants further study, but it is plausible that effective suppression of prostate tumor progression that was not fully achieved by treatment with AR-targeted therapies might lead to better targeting approaches through Wnt/β-catenin inhibition by RORα1 peptide therapies. In conclusion, our findings have provided evidence for the cross-regulation of RORα and the Wnt/β-catenin signaling pathway, and represent a robust approach integrating genome-wide attenuating responses with particularly pathologically and physiologically relevant pathways. Defining this crosstalk between RORα and the Wnt/β-catenin signaling axis may contribute further approaches to improve therapeutic strategies for treating specific human cancers.

## 4. Materials and Methods

### 4.1. Reagents

The following antibodies were purchased from Santa Cruz Biotechnology: Anti-β-catenin (sc-7963, 1:5000), Cyclin D1 (sc-8396, 1:3000), c-jun (sc-1694, 1:1000), c-myc (sc-40, 1:5000), Lamin A/C (sc-376248, 1:1000), tubulin (sc-5274, 1:5000), RORα1 (sc-26377, 1:1000), and common RORα (sc-6062, 1:1000). The following commercially available antibodies were used: Anti-FLAG antibodies (Sigma, Millipore-Sigma, St. Louis, MO, USA, F3165, 1:5000), anti-β-catenin (Sigma, A5316, 1:5000), and anti-RNA Polymerase II antibodies (Berkeley Antibody Company, Berkeley, CA, USA, 1:1000). LiCl was purchased from Sigma and added at 10 mM for 24 h before cell harvest.

### 4.2. Mice and Preparation of Primary Mouse Embryonic Fibroblasts

A pair of the heterozygous mice having the *staggerer* mutation (C57BL/6J-ROR^sg/+^) were purchased from the Jackson Laboratory and housed in the animal facility of the Sookmyung Women’s University according to standards of the Association for Assessment and Accreditation of Laboratory Animal Care (SNU-110324-3, 24 March 2011). The animals were genotyped by PCR. Genomic DNA was extracted from tail biopsies and amplified in two sets of reactions, one for each allele. The *staggerer* allele primers (5′–3′) were: CGTTTGGCAAACTCCACC and GTATTGAAAGCTGACTCGTTCC. The + allele primers were: TCTCCCTTCTCAGTCCTGACA and TATATTCCACCACACGGCAA. The amplified fragments (318 bp for + allele and 450 bp for *sg* allele) were detected by electrophoresis on agarose gel. Homozygous *Rora^sg/sg^* mice and their *Rora^+/+^* littermates were obtained by crossing heterozygous male and female breeders. The mice were weaned at four weeks of age. Male *Rora^+/+^* and *Rora^sg/sg^* mice were used in the experiment. Mouse embryonic fibroblasts were generated from a litter of embryos on embryonic day 13.5. Briefly, embryos were taken out aseptically and the head and liver were carefully removed from the isolated fetus, minced and trypsinized for 30 min at 37 °C. Cells were harvested, resuspended in Dulbecco’s modified Eagle’s medium supplemented with 10% fetal bovine serum, and plated on a 10 cm dish for each embryo.

### 4.3. GST Pull-down Assays

To examine the effect of RORα constructs on the binding to β-catenin, we first prepared GST β-catenin bound to Glutathione-Sepharose beads. The beads were incubated with the isolated RORα proteins in a buffer containing 20 mM Tris-HCl (pH 7.5), 150 mM NaCl, 0.2% Nonidet P40, and 10% glycerol. After extensive washing, the bound materials were subjected to Western blot analysis.

### 4.4. Tissue Array and Immunohistochemistry

Tissue array (SuperBioChips, Seoul, Korea) was stained as previously described [39]. The methods were carried out according to the relevant guidelines and regulations. The informed consents to use the tissue specimens for research purposes were obtained from patients, and the utilization of the specimens for this research was authorized and approved by the Institutional Review Board of the College of Medicine, Seoul National University (IRB No. 0608/001-003, 25 August 2006). All experiments were carried out according to approved guidelines.

### 4.5. Chromatin Immunoprecipitation (ChIP)

The ChIP was conducted in *Rora^sg/sg^* MEFs as previously described [40,41] using sheared fragments with an average size of approximately 300–500 bps. For PCR, 1 µL from 50 µL DNA extract and 25–30 cycles of amplification were used. The following primers (5′–3′) were used: *cyclin D1* promoter sense strand CCGGGCTTTGATCTTTGCTTA, antisense strand TCTGCTGCTCGCTGCTACTG.

### 4.6. Real-Time Q-PCR

The abundance of mRNA was detected by an ABI prism 7300 system with SYBR Green (molecular probes). Primer pairs were designed to amplify 90–150 bp mRNA specific fragments and confirmed as a unique products by melting curve analysis. The PCR conditions were 95 °C (5 min) and 40 cycles of 9595 °C (30 s), 5695 °C (30 s), and 7295 °C (30 s). The quantity of mRNA was calculated using ΔΔ*C*t method and normalized by using primers to detect β-actin or HPRT. All reactions were performed as triplicates. Primers (5′–3′) were: mCyclinD1, AACTACCTGGACCGCTTCCT and CCACTTGAGCTTGTTCACCA; mc-jun, TGAAAGCTGTGTCCCCTGTC and ATCACAGCACATGCCACTTC; mc-myc, TGAGCCCCTAGTGCTGCAT and AGCCCGACTCCGACCTCTT; mMcp-1, GGCTCAGCCAGATGCAGTTAAC and AGCCTACTCATTGGGATCATCTTG; mβ-actin, TAGCCATCCAGGCTGTGCTG and CAG GATCTTCATGAGGTAGTC; hRORα1, CGGTGCGCAGACAGAGCTAT and CCACAGATCTTGCATGGAATAATT; hCyclin D1, CTACTACCGCCTCACACGCTT and GGCTTGACTCCAGCAGGGCT; hc-Jun, GTCCACGGCCAACATGCTCA and TGTTTGCAACTGCTGCGTTAG; hc-Myc, CAGCTGCTTAGACGCTGGATT and GTAGAAATACGGCTGCACCGA; hHPRT, TGACACTGGCAAAACAATGCA and GGTCCTTTTCACCAGCAAGCT.

### 4.7. Cell Proliferation Assay and BrdU Incorporation Assay

LNCaP, RORα1-LNCaP, and RORα4-LNCaP cells (3 × 10^4^ cells each) were used in triplicates. Assay was performed as previously described [42]. To detect incorporated BrdU, rat monoclonal anti-BrdU antibody (Cat# OBT-0030, 1:200, Oxford Biotechnology, Raleigh, NC, USA) and Alexa Fluor 568-conjugated goat anti-mouse IgG (Molecular Probes, Eugene, OR, USA) were used.

### 4.8. Matrigel Invasion Assay

LNCaP cells stably expressing RORα1, RORα4, or RORα4+1NTD were used in Matrigel invasion assays along with control cells. Matrigel invasion assay was conducted as previously described [39]. Cultured cells were pretreated with 10 mM LiCl for 24 h, and 2.5 × 10^4^ LNCaP cells were loaded onto the top of a 24-well Matrigel invasion chamber assay plate (BD Biocoat, BD Biosciences, Bedford, MA, USA). Conditioned RPMI1640 medium containing 15% fetal bovine serum was added to the bottom chamber as a chemoattractant. After 22 h incubation, the cells that had migrated to the lower chamber of the filter were fixed with 100% methanol, stained with Giemsa, and quantified by counting the total number of cells in four different fields. All experimental studies were performed according to the manufacturer’s protocols. Values were expressed as means ± standard deviations for at least three independent experiments.

### 4.9. Cell Transformation Assay

Anchorage-independent growth of LNCaP, LNCaP-RORα1 and LNCaP-RORα4 was determined by analyzing cellular growth in semisolid medium. Cells (10^5^) were placed in Iscove’s media containing 0.4% noble agar containing 10% FCS. Cells were allowed to grow for three weeks in 5% CO_2_, and the formation of colonies containing >50 cells was analyzed. Colonies were counted in ten different fields, and the total colony number/well was calculated. The representative image is shown for each group.

### 4.10. Tumorigenicity Assay

For experiments examining tumor formation in vivo, a total of ten million cells with an equal volume of Matrigel (BD Biosciences, Bedford) was injected subcutaneously at the left flank into three groups of 6-week-old athymic *nu*/*nu* male mice (Orient, Seoul, Korea). Tumors were measured weekly and the experiment was terminated when the largest tumor was about 10 mm in diameter. Tumors were excised and weighed. Statistical differences in tumor weights were determined by a Student’s t-test using the Statview package (Abacus Concepts, Inc., Berkeley, CA, USA). These experiments were carried out with the approval of the Institutional Animal Care and Ethics Committee.

### 4.11. Quantification and Statistical Analysis

Values were expressed as mean ± SD or SEM. Significance was analysed by a one-tailed, unpaired t-test or one-way ANOVA using GraphPad Prism software. *p* < 0.05 was considered statistically significant. * *p* < 0.05, ** *p* < 0.01, *** *p* < 0.001.

## Supplementary Materials

Supplementary materials can be found at https://www.mdpi.com/1422-0067/20/7/1684/s1.

## Abbreviations

RORα	Retinoid acid-related orphan receptor α
ONR	Orphan Nuclear Receptor
NTD	N-terminal domain
DBD	DNA-binding domain
LBD	Ligand-binding domain
TCF	T cell factor
LEF	lymphoid enhancer factor
CKI	Casein kinase I
GSK-3β	Glycogen synthase kinase-3β
Dvl	Dishevelled
MEF	Mouse embryonic fibroblast
ChIP	Chromatin immunoprecipitation
