# Supplementary material for: N-Terminal Domain Mediated Regulation of RORα1 Inhibits Invasive Growth in Prostate Cancer
Source: Int J Mol Sci. 2019 Apr 4;20(7):1684. doi: 10.3390/ijms20071684 (PMC6479703; doi:10.3390/ijms20071684)
Supplement: Supplementary file 1 [file ijms-20-01684-s001.pdf]

Supplemental Data

N-terminal Domain Mediated Regulation of RORα1 Inhibits Invasive Growth in Metastatic Prostate Cancer

Su Chan Park, Il Geun Park, Hyunkyung Kim and Ji Min Lee

Supplemental Data include 1 Supplemental figure, 3 Supplemental Tables, Supplemental Experimental Procedure, and Supplemental References.

SUPPLEMENTAL FIGURE S1

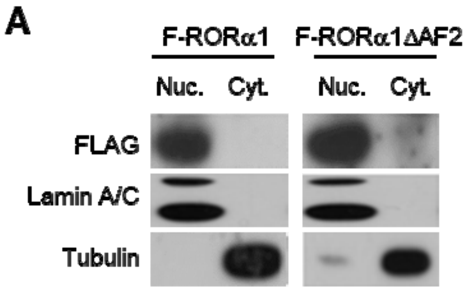

(**A**) Cytoplasmic and nuclear fractions of FLAG-RORα1 and RORα1ΔAF2 expressing 293T cells were isolated, the concentration of nuclear and cytoplasmic protein was measured by Bradford assay, the same amount of nuclear and cytoplasmic protein was subjected to SDS gel, and immunoblots were performed with anti-FLAG, Lamin A/C, and tubulin antibodies.

SUPPLEMENTAL FIGURE S2

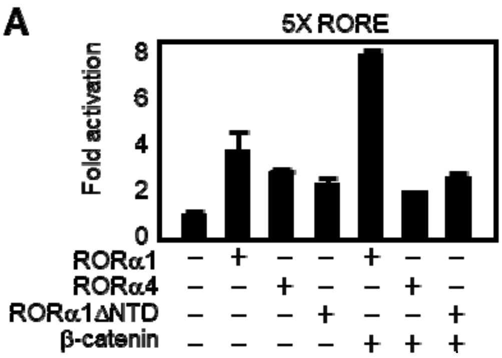

Figure S2. β-Catenin functions as a selective coactivator for RORα1.

(**A**) Luciferase assay was conducted after transfection of β-catenin with RORα1, RORα4, or RORα1ΔNTD on a 5X RORE luciferase reporter. Values are expressed as mean ± SD for three independent experiments.

SUPPLEMENTAL FIGURE S3

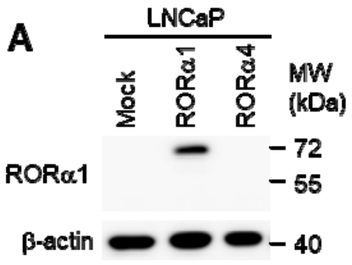

Figure S3. RORα1-specific antibody selectively detects RORα1-expressing LNCaP cells.

(**A**) Immunoblotting and analyses of RORα1 specific antibody in RORα1- and RORα4-expressing LNCaP cells. This antibody detects RORα1 with RORα1-expressing LNCaP cells, not with RORα4-expressing LNCaP cells.

SUPPLEMENTAL FIGURE S4

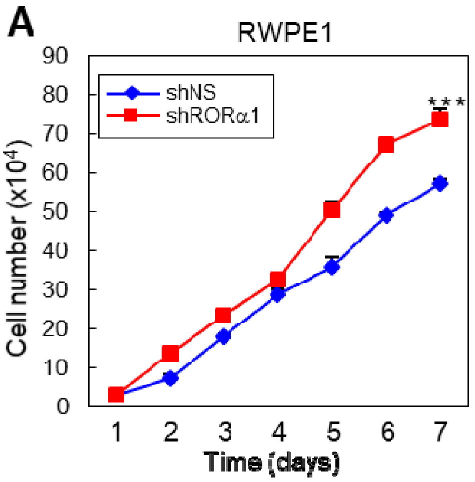

Figure S4. Knockdown of RORα1 increases the proliferation of prostate cancer cells.

(**A**) Proliferation curves of nonspecific shRNA (shNS) or RORα1 shRNA (shRORα1)-expressing RWPE1 cells. Values are represented as mean ± SD for three independent experiments. (*** $p<0.001$).

SUPPLEMENTAL EXPERIMENTAL PROCEDURES

Retroviral infection

pBabe-puro and pBabe-puro-RORα1 vectors were used to produce retrovirus, as previously described [1]. Proliferating cells (30-50% confluent) were infected for 8 hr on two successive days, with a 16 hr interval and medium change between infections. After 48 hr, cells were selected in puromycin (2 μg/mL) for 4-5 days and expanded in antiboiotic-free medium. Lysates were obtained 14 days after infection.

Generation of expression constructs

To generate the FLAG- or GFP-tagged plasmids expressing RORα, p3XFLAG-CMV10 (N-terminal 3XFLAG fusion vector) and pEGFP-C1 (C-terminal GFP fusion) vectors were used. The restriction sites for *EcoRI* and *BamHI* were introduced by PCR to the 5'- and 3'-regions of RORα, respectively. The PCR products were cut out by *EcoRI* and *BamHI* and ligated into p3XFLAG-CMV10 and pEGFP-C1 vectors that had been treated with the same restriction enzymes. All constructs were verified by restriction enzyme analysis and DNA sequencing.

Reporter assays

Reporter plasmid 5XRORE-tk-luciferase was gifts from by G. E. O. Muscat (University of Queensland). A reporter plasmid for RORα-mediated transcriptional regulation was generated by incorporating five tandem copies of ROR response element (5'-TATATCAAGGTCAT-3') into the luciferase reporter plasmid pTK-luc [2]. Luciferase activity was measured in a luminometer 48h after transfection and normalized to β-galactosidase expression with a luciferase assay system (Promega). Values are expressed as means ± standard deviations for at least three independent experiments.

SUPPLEMENTAL REFERENCE

1. Rubio, M.A.; Kim, S.H.; Campisi, J. Reversible manipulation of telomerase expression and telomere length. Implications for the ionizing radiation response and replicative senescence of human cells. *J. Biol. Chem.* **2002**, *277*, 28609-28617.
2. Medvedev, A.; Yan, Z.H.; Hirose, T.; Giguère, V.; Jetten, A.M. Cloning of a cDNA encoding the murine orphan receptor RZR/ROR gamma and characterization of its response element. *Gene.* **1996**, *181*, 199-206.
